# The potential of melatonin and its crosstalk with other hormones in the fight against stress

**DOI:** 10.3389/fpls.2024.1492036

**Published:** 2024-12-05

**Authors:** Lina Xu, Yafei Zhu, Yakun Wang, Luyan Zhang, Lijie Li, Ley Juen Looi, Zhiyong Zhang

**Affiliations:** ^1^ Henan Institute of Science and Technology, School of Agriculture, Xinxiang, Henan, China; ^2^ Kaifeng Meteorological Service, Agricultural Meteorological Observation Station, Kaifeng, Henan, China; ^3^ Henan Institute of Science and Technology, School of Life Sciences, Xinxiang, Henan, China; ^4^ Faculty of Forestry and Environment, Department of Environment, Universiti Putra Malaysia, Serdang, Selangor, Malaysia

**Keywords:** melatonin, environmental stresses, biosynthesis, phytohormones, stress response

## Abstract

Climate change not only leads to high temperatures, droughts, floods, storms and declining soil quality, but it also affects the spread and mutation of pests and diseases, which directly influences plant growth and constitutes a new challenge to food security. Numerous hormones like auxin, ethylene and melatonin, regulate plant growth and development as well as their resistance to environmental stresses. To mitigate the impact of diverse biotic and abiotic stressors on crops, single or multiple phytohormones in combination have been applied. Melatonin is a multifunctional signaling molecule engaged in the development and stress response of plants. In the current review, we discuss the synthesis and action of melatonin, as well as its utilization for plant resistance to different stresses from the perspective of practical application. Simultaneously, we elucidate the regulatory effects and complex mechanisms of melatonin and other plant hormones on the growth of plants, explore the practical applications of melatonin in combination with other phytohormones in crops. This will aid in the planning of management strategies to protect plants from damage caused by environmental stress.

## 1 Introduction

Melatonin (MT), or N-acetyl-5-methoxytryptamine, is an indoleamine first isolated from the pineal gland of cattle in 1958 (Lerner et al., 1958). It regulates rhythmic behaviors in humans and other mammals (Lerner et al., 1959). Since its discovery in plants in 1995, the study of MT has become one of the fastest-growing areas in plant physiology. In plants, MT acts as a growth promoter and antioxidant, regulating growth and development and various physiological processes. MT promotes crop seed germination, root organ development, biomass accumulation, and meristematic growth, and it also plays a role in reproductive development and leaf senescence (Kumari et al., 2023). Similarly, MT promotes cell division and elongation, thus contributing to increased biomass and production. Plants produce endogenous MT *in vivo*, present in various plant tissues such as seeds, roots, leaves and fruits. This endogenous MT plays a crucial function in the regulation of plant development and the response to stress. Studies have shown increased MT accumulation in plant tissues during root development, as well as under conditions of drought, salinity and temperature extremes, suggesting the importance of MT in enhancing plant adaptation to environmental stresses.

Since higher MT levels in plants can promote growth, and some plants have a limited capacity to produce MT, they may benefit from the application of exogenous MT. For example, cotton seeds pretreated with MT (20 µM) achieved better germination by increasing antioxidant enzymes and inhibiting ABA levels in the seeds (Xiao et al., 2019). Similarly, seeds of stevia soaked in 20 mM MT showed enhanced morphological development (more weight gain and increased number of leaves) by modulating the levels of phenolic compounds (Simlat et al., 2018). The application of exogenous MT not only improves seed germination but also promotes root organ development for better soil nutrient uptake, and the positive effects of MT on root development have been documented in several plant species. Specifically, 10-30 µM MT significantly enhanced root system vigor, root hair growth, and lateral root development through root irrigation (Tian et al., 2024). Exogenous MT induced the germination of root primordia in the epidermal cells of the winged bean root, altering the length and number of adventitious and lateral roots (Arnao and Hernández-Ruiz, 2007). By improving root development, MT promotes improved growth and nutrient uptake in plants such as cucumber (Xu et al., 2024), soybean (Wang et al., 2021a) and *Arabidopsis thaliana* (Yang et al., 2021). In addition to acting as a growth promoter, MT also scavenges reactive oxygen species (ROS) and free radicals, protecting plants from various environmental coercion. For instance, externally applied MT has been linked to increased plant resilience to stressors such as high temperature, drought, and saline conditions (Reiter et al., 2022; Khanna et al., 2023). Moreover, MT improves plant immunity and resistance to biotic stresses. Studies have demonstrated that the localized application of 0.05-0.5 mM MT increases the resistance of apples to apple scab disease (Yin et al., 2013). Higher levels of MT enhanced Arabidopsis’ resistance to Botrytis cinerea infection (Zhu et al., 2021).

As mentioned above, aspects such as the endogenous role of MT in regulating stress response, the limited capacity of plants to produce higher internal MT (genetic variation) and the consequent susceptibility to stress are discussed. Although the stress-responsive regulatory role of MT has been extensively studied, the mechanism of crosstalk between MT and other phytohormones and plant growth regulators under environmental stress has not yet been clarified. A significant amount research is still needed to address the complex regulatory network between MT and phytohormones to maintain plant growth and adapt to the environmental conditions. In recent years, due to climate change, there has been an increasing need to protect crops from heat, drought, and salinity. For this reason, it is particularly relevant to describe the crosstalk mechanisms of MT with other plant hormones and plant growth regulators under stressful environment.

## 2 Melatonin biosynthesis

**Figure 1:**
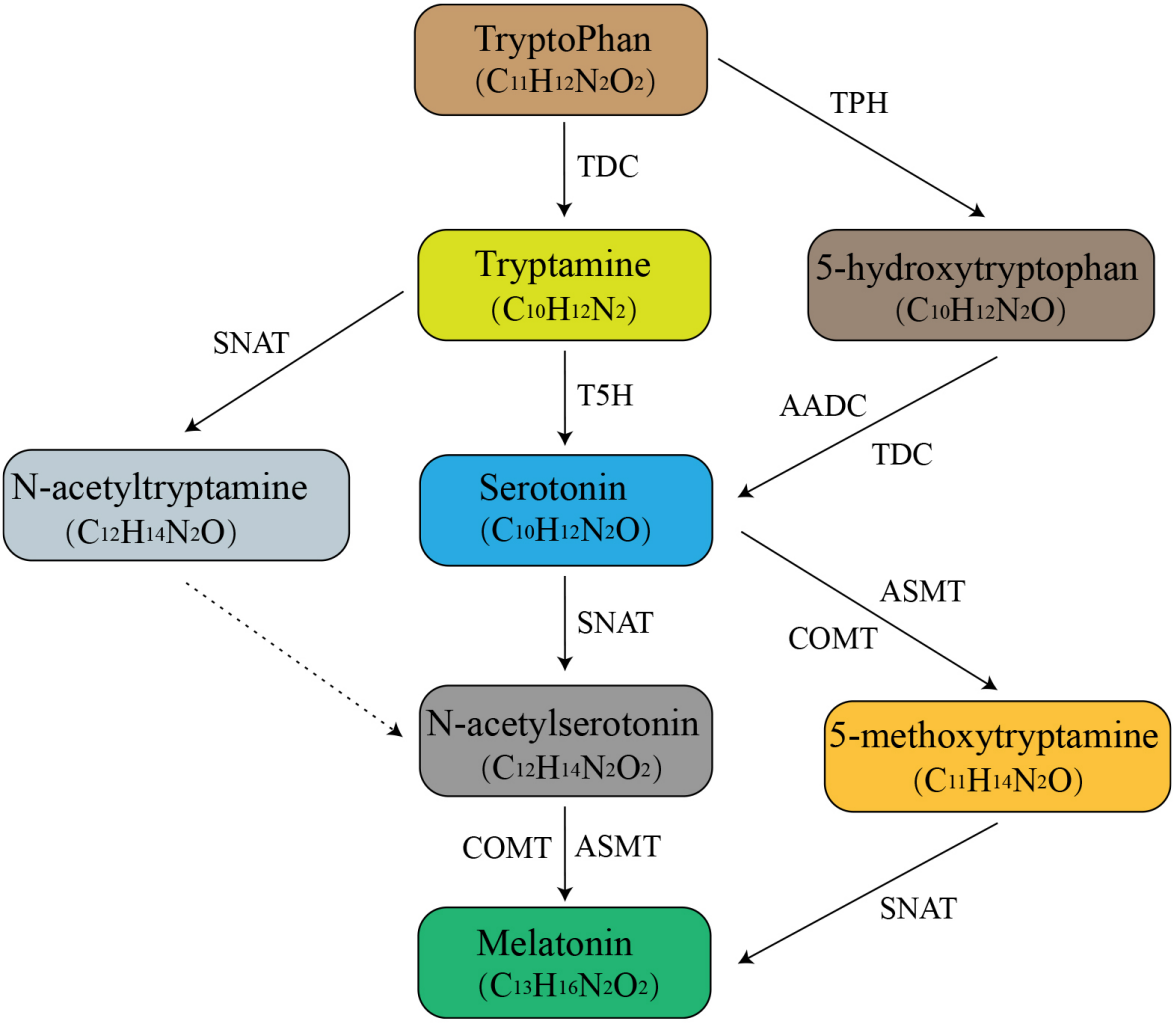
The biosynthetic pathway of MT in plants. Arrows with a solid line represent MT biosynthetic pathways, while the one with a dotted line represents speculative or potential steps. TDC, tryptophan decarboxylase; TPH, tryptophan hydroxylase; T5H, tryptamine 5-hydroxylase; SNAT, serotonin-*N*-acetyltransferase; ASMT, *N*-acetylserotonin methyltransferase; AADC, aromatic amino acid decarboxylase; COMT, caffeic acid *O*-methyltransferase.

In plants, MT biosynthesis involves a series of enzymatic reactions, with serotonin serving as a key intermediate. The synthesis process involves six enzymes including tryptophan decarboxylase (TDC), tryptophan hydroxylase (TPH), tryptamine 5-hydroxylase (T5H), serotonin-*N*-acetyltransferase (SNAT), *N*-acetylserotonin methyltransferase (ASMT) and caffeic acid *O*-methyltransferase (COMT). Synthesis begins with the induction of tryptophan (Trp) decarboxylation by TDC to form tryptamine (Zhou et al., 2020), after which 5-hydroxytryptophan is formed by hydroxylating T5H at the indole ring’s 5-position, commonly known as serotonin. Serotonin is then converted to N-acetylserotonin by SNAT, which in turn generates MT via an ASMT/COMT-catalyzed reaction (**Figure 1**).

Under certain environmental stresses, plants produce MT by an alternative pathway involving 5-hydroxytryptophan or 5-methoxytryptophan or both as intermediates. In this process, tryptophan is catalyzed by TPH to form 5-hydroxytryptophan. For example, during senescence, plants produce large amounts of serotonin, and the synthetic pathway involves the metabolism of serotonin to 5-methoxytryptamine via ASMT/COMT. Subsequently, 5-methoxytryptamine is converted to MT by acetylation of SNAT (Arnao and Hernández-Ruiz, 2015; Tan et al., 2015; Back et al., 2016; Tan and Reiter, 2020; Back, 2021; Colombage et al., 2023), following tryptophan/tryptamine/serotonin/5-methoxytryptamine/melatonin pathway. SNAT catalyzes the final step of MT biosynthesis, which occurs in chloroplasts, while ASMT/COMT participates in cytoplasmic terminal reactions (Back et al., 2016). Both chloroplasts and mitochondria may be involved in the synthesis of melatonin in plants. Under normal conditions, chloroplasts are the main site of melatonin production in plants. However, if the normal process is disrupted, such as by inhibiting the transcription of tryptamine 5-hydroxylase, the main melatonin synthesis pathway will be shifted from chloroplasts to mitochondria (Sang-Kyu et al., 2013).

## 3 Effects of melatonin on plant growth and development

**Figure 2:**
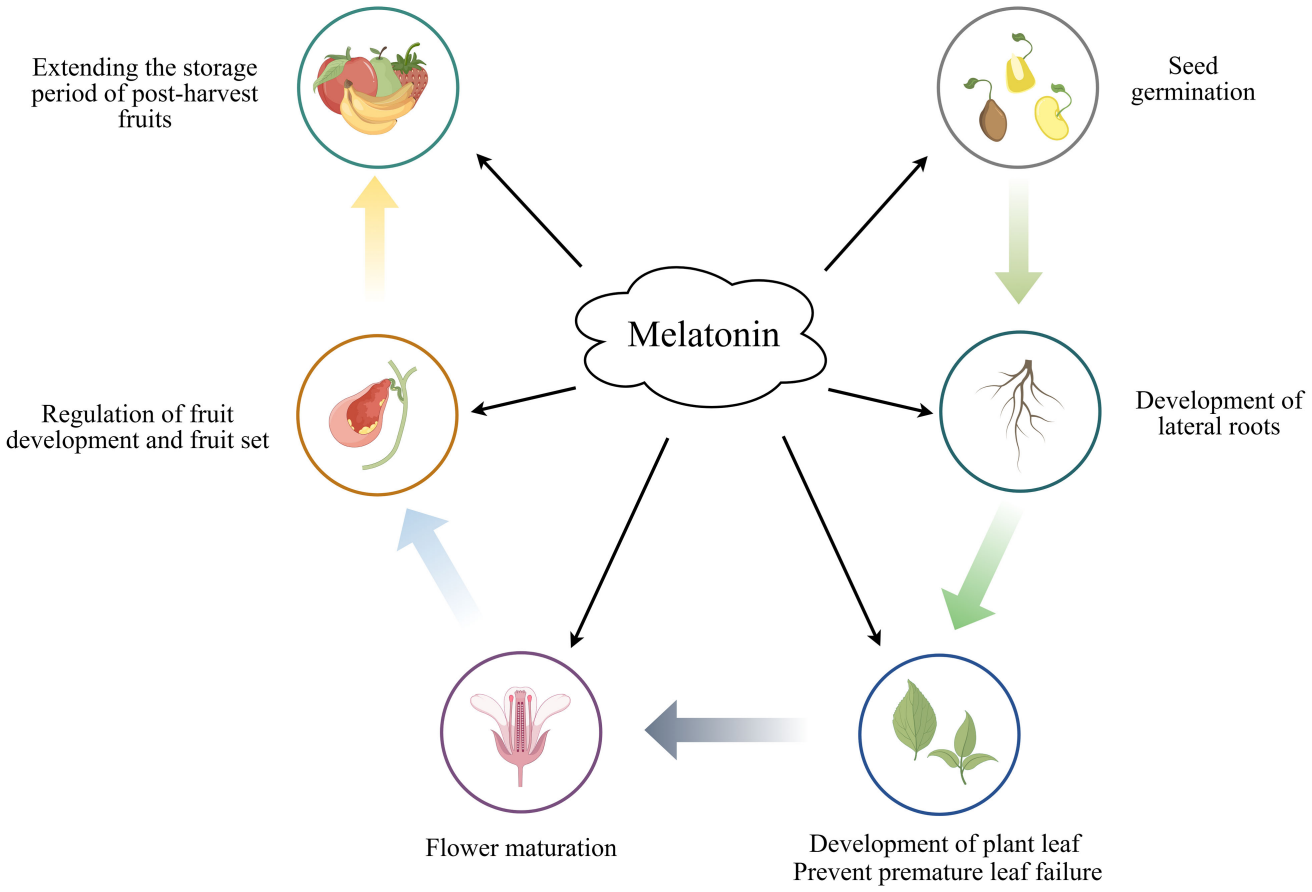
The role of melatonin in plant growth and development.

The complex role of melatonin in different aspects of plant physiology including seed germination, root development, stem elongation, flowering and fruit ripening (Wang et al., 2022b) (**Figure 2**). MT has been reported to accelerate the germination of different plant seeds, thereby improving their productivity and survival. Bai et al. (2020) found that pretreatment of cotton seeds with MT increased the number of stomata and facilitated pore opening, increased starch metabolism and osmotic substance content, and generally improved the germination rate of cotton seeds. Yan et al. (2021) showed that triggering aged oat seeds with MT may repair the cellular ultrastructure by restoring membrane integrity, and the relatively intact cellular structure may synthesize a variety of amino acids to provide energy for seed germination, thereby improving the germination rate. Furthermore the exogenous application of MT has been shown to increase root growth, tiller number, spike number, photosynthetic rate, and carbon assimilation capacity in winter wheat (Ye et al., 2020). Qiao et al. (2019) found that exogenous MT treatment of wheat seedlings under nitrogen-deficient conditions promoted N uptake and assimilation, as well as plant growth and yield, through the up-regulation of N uptake and metabolism-related enzyme activities. Li et al. (2024) demonstrated that the application of melatonin as a seed dressing could improve photosynthetic efficiency, enhance the activity of carbon and nitrogen metabolic enzymes, facilitate material transport, and ultimately increase both the number of effective pods and the yield of peanuts across varying nitrogen levels.

MT prevents chlorophyll loss by acting as an antioxidant in plants and protects against ROS-induced senescence. In addition to favoring chlorophyll protection, the photosynthetic function of chlorophyll is improved through the maintenance of redox homeostasis (Sharif et al., 2018). MT has been found to delay cucumber leaf senescence and increase its yield by regulating chlorophyll degradation as well as photoinhibition (Liu et al., 2022). Arnao and Hernández-Ruiz, (2009) found that MT exerted a protective effect on chlorophyll degradation during the senescence of barley leaves, mainly because MT acted as antioxidant, detoxified ROS/RNS, and slowed down the leaf senescence process. In melatonin-treated tomato leaves, the up-regulation of leaf chlorophyll degradation genes and senescence genes induced by methyl jasmonate was inhibited, thereby slowing the leaf senescence process (Wang et al., 2019).

The fruit ripening process in plants is similarly regulated by MT. Strawberries treated with exogenous MT injection showed higher total phenol content, increased expression of PALase activity genes and slowed down the ripening of the fruits (Mansouri et al., 2023b). Additionally, exogenous MT treatment of post-harvest cherries reduced the incidence of decay and respiration rate, while also slowing down the degradation of firmness, brightness and soluble solids content, thereby maintaining better fruit quality (Wang et al., 2019). Furthermore, MT reduces post-harvest decay of strawberry fruits by increasing the content of total phenolics and anthocyanins, enhancing ATP levels and boosting antioxidant activity (Aghdam and Fard, 2017b).

## 4 Melatonin’s function under abiotic stresses

The majority of plants encounter abiotic stressors which in turn cause stress that affects their growth. The main abiotic stress factors include temperature extremes (heat, cold and freezing), flooding, drought, heavy metals and high soil salinity (Zhang et al., 2022). As a significant biomolecule, MT is essential for controlling how plants develop and adapt to a variety of abiotic stressors. This regulation is achieved by modulating several common and stress-specific reactions (**Table 1**). Findings from transcriptome analysis have shown the expression patterns of MT synthesis-related genes (COMT, TDC, T5H, SNAT and ASMT) under various abiotic stresses (Ahn et al., 2021; Xing et al., 2021). Additionally, it has been found that MT application modulates a variety of physiological processes and increases endogenous MT content to enhance plant tolerance (Fan et al., 2018b). The following sections will discuss how MT regulates stress tolerance in plants in response to different types of abiotic stress.

**Table 1:** The impact of MT on various stresses and different crops.

Plant	Application concentration	Application method	Stresses	Effect	References
Cucumber seedlings	30-40μM	foliar spray	cold stress	Controls metabolism and regulates antioxidant enzyme activity	(Uzal et al., 2022)
Tea seedlings	150μM	folia spray	Cadmium stress	Increase soluble protein and photosynthetic pigment accumulation, regulate soil enzyme activities to inhibit Cd uptake by roots	(Tan et al., 2022)
Wheat	100μM	foliar spray	Flood stress	Regulates antioxidant enzyme activity, acceleration of anaerobic respiration, reduction of MDA content, O_2_^-^, PDA, LDH and ADH activities	(Ma et al., 2022)
Collard greens	50μM	soak seeds	salt stress	Reduction of superoxide and hydrogen peroxide production and regulation of antioxidant enzyme activity	(Mohamed et al., 2020)
Cotton	25μM	soak seeds	salt stress	Up-regulation of the number of genes encoding antioxidant enzymes and significant differential expression of genes in a large number of phytohormone signaling pathways	(Zhang et al., 2021b)
Rice	100μM	soak seeds	drought stress	Regulates antioxidant enzyme activity and soluble protein content and reduction of malondialdehyde content	(Li et al., 2022b)
Corn	200μM	rooting	drought stress	Improvement of leaf water properties	(Gul et al., 2022)
Tomato	100μM	foliar spray	heat stress	Increasing endogenous MT levels and photosynthetic pigment content and up-regulating their biosynthetic gene expression	(Jahan et al., 2021a)
Wheat	100μM	foliar spray	heat stress	Reduces lipid peroxidation content, increases Pro biosynthesis, activates the ascorbate-glutathione (ASC-GSH) cycle, and increases glutathione reductase (GR) activity	(Buttar et al., 2020)
Melons	100μM	sprinkler	heat stress	Significantly higher levels of FaTHsfA2a and HSP90 mRNA induced antioxidant mechanisms, low strawberry leaf H_2_O_2_ production and lipid peroxidation, and improved membrane stability	(Manafi et al., 2021)
Rice seed	100μM	soak seeds	heat stress	Increased germination potential of rice seeds; increased stem and root length; decreased malondialdehyde content	(Yan et al., 2021)
Melon seed	10-50μM	soak seeds	salt stress	Increased seed germination	(Luis Castañares and Alberto Bouzo, 2019)

### 4.1 The function of MT in response to water stress

Water stress causes an imbalance in the distribution of water in plants, disrupting cellular homeostasis and severely inhibiting plant growth. Bioenergy production from photosynthesis is inhibited by limiting the uptake and transfer of light energy by reducing chlorophyll content, actual photochemical efficiency, and photochemical burst. Because of the water shortage under drought stress, plants release a lot of ROS, which leads to chloroplast damage and inhibition of photosynthesis by decreased chlorophyll content (Abid et al., 2018; Imran et al., 2021). MT treatment reduced the inhibitory effects of drought stress on photosynthesis and biomass accumulation. He et al. (2023) showed that MT (100 µM) significantly increased stomatal conductance of sugar beet leaves under drought stress, promoting leaf gas exchange, enhancing chlorophyll content, and facilitating photosynthesis. For instance, the increased expression of endogenous MT synthesis genes (MdTDC1, MdAANAT2, MdT5H4, and MdASMT1) induced an increase in MT synthesis in drought-stressed Begonia, which regulates water homeostasis to protect the plant from injury (Khattak et al., 2023). Studies also found that exogenous MT can mitigate the effects of ROS on crops, such as maize seedlings (Muhammad et al., 2023) and rice (Li et al., 2022a), by regulating the antioxidant defense system, thereby increasing the resilience of plants to drought.

Flooding is also harmful to the development and growth of plants, primarily through restricting gas diffusion, which causes anaerobic respiration within the roots and encourages the build-up of ROS (Wu et al., 2021). In the event of flooding, MT can alleviate the stress caused by flooding. For instance, exogenous MT treatment of kiwifruit plants effectively mitigate damage from flooding, primarily because treated kiwifruit roots had reduced ROS accumulation and increased antioxidant activity throughout the stressful period, helping to alleviate stress (Huo et al., 2022). Wheat is the most widely grown staple food crop (Chen et al., 2024). In a study, it was found that spraying MT (100 µM) both prior to and following flooding, could protect wheat roots from flooding-induced oxidative damage by up-regulating antioxidant enzymes and maintaining leaf photosynthesis. MT-treated plants had a superior water status and less oxidative harm than flooded plants, thereby improving wheat flooding tolerance by enabling it to maintain a higher photosynthetic capacity (Ma et al., 2022).

### 4.2 The function of MT in response to salt stress

Nowadays, salt stress has become a worldwide issue, with an increasing number of crops suffering from yield loss due to salt stress affecting their quality. According to recent research, exogenous MT treatment can improve salt tolerance in plants (Nawaz et al., 2021). First, exogenous application of MT can increase the germination rate of plant seeds. For instance, during seed germination, MT by modulating energy synthesis and ion homeostasis in seeds during salt stress, improves germination of seeds and seedling growth (Wang et al., 2024). In many crops such as bitterbrush (Li et al., 2019b), cotton (Chen et al., 2020) and wheat (Wang et al., 2022a), seeds pretreated with exogenous MT have higher germination rates under salt stress.

Due to the high concentration of salt in the soil, the ionic homeostasis of plants was disturbed, resulting in a large accumulation of ions causing cell damage. However, MT as a salt stress ameliorator could regulate the ionic homeostasis and prevent the accumulation of ions in the cells, which was mainly attributed to the fact that MT regulated the uptake and transportation of ions (Zhan et al., 2019). A study found that MT significantly up-regulated two important ion channels (NHX1 and AKT1) under salt stress, promoting tolerance to salinity and playing a crucial role in maintaining ion homeostasis (Li et al., 2012). Exogenous MT application improved the salt tolerance of potatoes by increasing the K^+^/Na^+^ ratio, increasing the K^+^ content, and decreasing the Na^+^ and Cl^-^ content (Yu et al., 2018). In addition, MT increases the accumulation of organic osmoregulatory substances to protect cells from dehydration and maintains ionic homeostasis to inhibit intracellular Na^+^ and Cl^-^ accumulation (Gao et al., 2019; Altaf et al., 2021). Furthermore, MT helps conserve plant water by regulating stomata and reducing water loss due to transpiration, while also stimulating root development under salt stress to improve the ability of plant roots to absorb water and alleviate drought caused by salinity (Duan et al., 2022; Khan et al., 2023).

Under salt stress, melon seedlings treated with 50 µM exogenous MT showed significantly better development and increased chlorophyll in their leaves compared to untreated plants (Luis Castañares and Alberto Bouzo, 2019). Combined foliar (100 µM) and root (100 µM) applications of MT up-regulated SNAT gene expression and increased endogenous MT levels in *Arabidopsis thaliana* and sugar beets, and enhanced photosynthesis, hydration status, ionic homeostasis, and antioxidant defense systems in sugar beets to increase their tolerance to salt (Zhao et al., 2018; Su et al., 2021; Zhang et al., 2021a). Similarly, blueberry plants treated with exogenous MT (200 µM) maintained significantly higher chlorophyll content, leaf thickness, and photosynthetic capacity under salt stress conditions (Jia et al., 2023). MT protects rice plants from salt damage during development by increasing leaf photosynthetic efficiency, total antioxidant capacity, and lutein cycling, as well as by increasing the estimate of the lutein pool to dissipate excess light energy (Yan and Mao, 2021).

### 4.3 The function of MT in response to extreme temperature

Plants experience heat stress during growth as a result of global warming and rising temperatures, which interferes with normal growth and development. High temperatures often lead to oxidative stress, causing oxidative damage to plants. MT acts as an important protective agent for plants against heat stress by enhancing the antioxidant system and reducing ROS production (Hassan et al., 2022). When heat stress is applied to plants, MT concentration increases significantly (Tal et al., 2011). For example, very high temperature (48°C) induced endogenous MT in apple plants by altering the expression levels of MdTDC1, MdAANAT2, and MdASMT9 synthase genes, especially MdASMT9, which showed the greatest increase in expression levels and alleviated high temperature-induced stress in apple plants (Rehaman et al., 2021). Heat stress up-regulates the expression of MT biosynthetic genes (TDC, T5H, SNAT and ASMT) in tomato seedlings (Jahan et al., 2021a). The application of 20 µM exogenous MT with irrigation water promoted the induction of ROS in tomato anthers under heat stress through up-regulating the activity of several antioxidant enzymes to protect pollen viability and fruit formation from heat damage, following MT application at high temperatures, pollen viability and germination increased by 45.7% and 30.5% (Qi et al., 2018).

Cold stress also negatively impacts plant development; low temperature induces cell membrane damage leading to a decrease in palpable mobility and disruption of ionic homeostasis. Cold stress adversely affects photosynthesis by disrupting the electron transport chains in mitochondria and chloroplasts resulting in the release of ROS (Fan et al., 2015; Shi et al., 2015). MT significantly improves photosynthetic efficiency under low-temperature stress conditions by enhancing the activities of key enzymes involved in carbon fixation, such as ribulose-1,5-bisphosphate carboxylase/oxygenase (Wang et al., 2020). MT plays a protective role during cold stress; for example, cold stress (4°C) scale up the level of MT biosynthesis genes (TDC, T5H, SNAT, ASMT), promoting the accumulation of endogenous MT and increasing its level in tomato plants to mitigate the damage caused by stress (Sharafi et al., 2019). In pepper seedlings under cold stress, Altaf et al. discovered that spraying MT (200 µM) up-regulated the biosynthetic gene expression of endogenous MT, which contributed to the support of high gloss and chlorophyll molecular integrity by increasing the levels of photosynthetic proteins, starch, sucrose, soluble sugars and glucose, thereby protecting plants from cold damage (Altaf et al., 2022). Altogether, these abilities of MT promote plant growth and survival under cold stress conditions. However, the exact mechanism of MT action in plants remains unclear, and further studies are needed to fully understand the functions and potential applications in agriculture.

### 4.4 The function of MT in response to heavy metals

In addition to its role in abiotic stress tolerance, MT has been found to act as a protective agent against heavy metal stress in plants. MT protects plants from heavy metal-induced damage by coordinating cellular heavy metal (HM) ions and improving nutrient and redox balance, osmotic adjustment, and primary and secondary metabolism (Hoque et al., 2021). Accumulation of metal ions leads to water loss in plant cells, while exogenous MT maintains osmotic balance by stimulating the production of osmoregulatory substances and stabilizing proteins and membranes (Fan et al., 2018a). Similarly, MT regulates plant tolerance to heavy metals, and plants mitigate oxidative stress caused by heavy metal exposure by stimulating the production of enzymatic antioxidants, minimizing oxidative damage to plant cells. Hoque et al. (2021) found that MT enhanced SOD activity, thereby reducing superoxide radical production and oxidative damage from exposure to heavy metals. In addition, MT enhanced the activities of antioxidant enzymes such as SOD, CAT and POD under HM stress. Plants also respond accordingly to mitigate the damage, for instance, under oxidative damage caused by heavy metals, chili pepper seedlings up-regulated genes related to key defenses (e.g., SOD, CAT, POD, GR, GST, APX, GPX, DHAR, and MDHAR) and MT biosynthetic genes. Exogenous MT supplementation also increased proline and secondary metabolite levels, and the up-regulation of their encoding genes enhanced the tolerance of pepper seedlings to heavy metal stress (Altaf et al., 2023). MT plays an important role in regulating the expression of genes related to HM detoxification and tolerance mechanisms. It was shown that MT isolated HMs and prevented their toxicity by upregulating the expression of metallothionein, a small metal-binding protein (Xu et al., 2020). Although MT has the potential to mitigate HM stress in plants, further studies on its mechanism of action are needed.

The above information summarizes the main functions of melatonin under different abiotic stresses and its regulatory roles in plant growth and development during stressful conditions. The regulatory effects of have been discussed in relation to abiotic stresses such as water stress, salt stress, extreme temperature, and heavy metal exposure. MT can regulate the water balance of plants, protect them from injury and improve stress tolerance. Under salt stress, MT helps maintain energy synthesis and ionic homeostasis, prevent cellular dehydration, enhances salt tolerance, and improves seed germination of plant seeds. Additionally, MT treatment protects the cell membrane integrity of plants during extreme temperatures, reducing cell damage caused by stress, and facilitating the process of photosynthesis. In short, when plants are under abiotic stress, MT can effectively alleviate the damage caused by various stressors, maintain normal growth activities through different ways, and protect the growth and development of plants.

## 5 Melatonin function under biotic stresses

**Figure 3:**
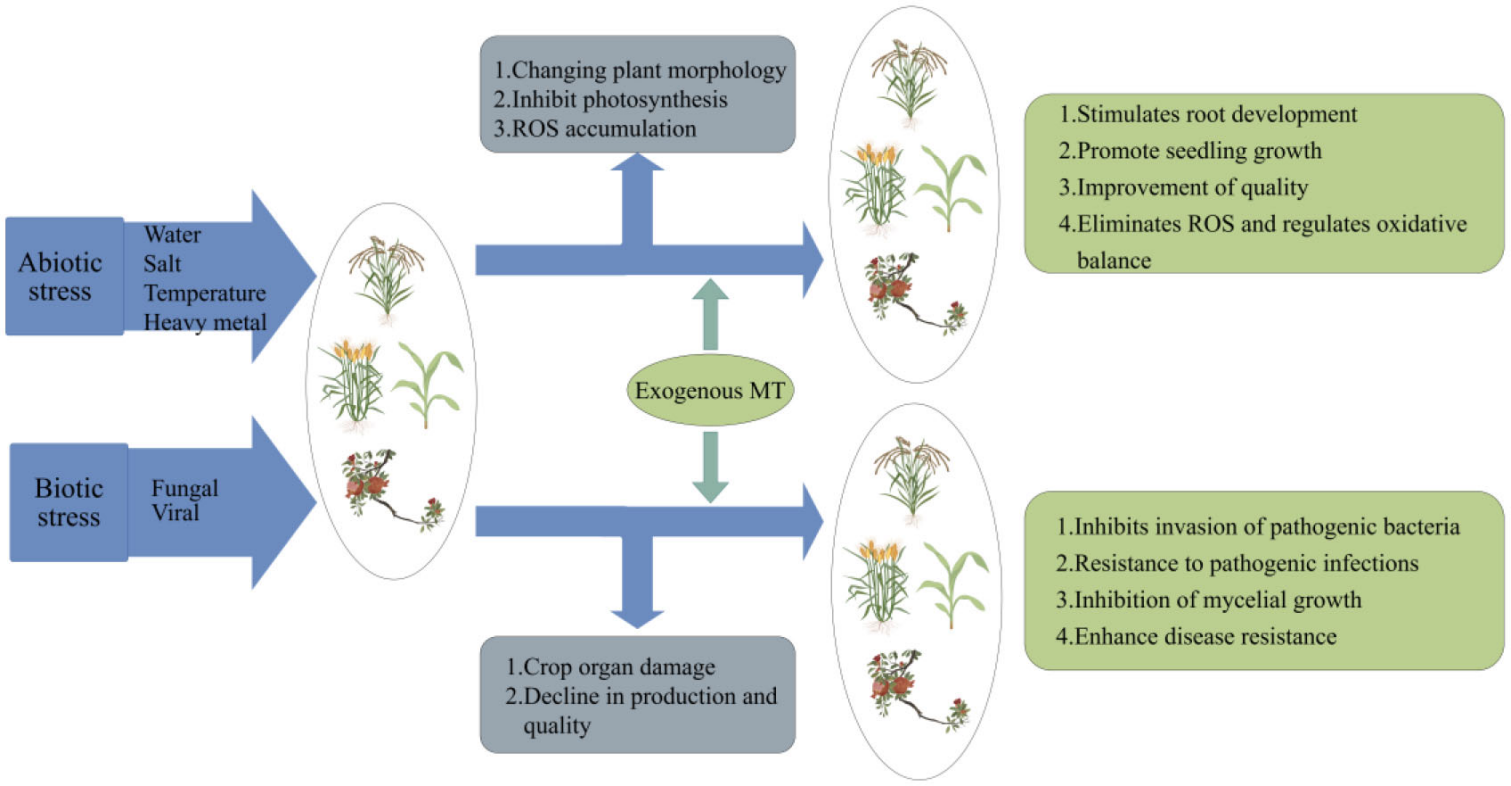
Crop damage under abiotic/biotic stresses and effects after exogenous application of MT.

Biotic stress refers to the harmful impacts of fungus, bacteria, viruses and other microbes on plants. When infected, these pathogens can damage various parts of plant, adversely affecting its quality and yield (Suzuki et al., 2014). Plants activate a highly developed immune system when subjected to biotic stress, starting with the waxy, thick cuticle and unique trichomes on their surfaces, which effectively prevent pathogens or insects from attaching to the plant (Moustafa-Farag et al., 2020; Yong-Sun and Sajid, 2022). Plants can identify pathogens through two different pathways: pattern recognition receptors (PRRs) like flagellin, which induce PAMP-triggered immunity (PTI), and plant resistance proteins, that recognize specific effectors, or non-toxic proteins from pests or pathogens. This recognition triggers a defense response called effector-triggered immunity (ETI) (Shahid et al., 2019; Khan et al., 2021). It has been shown that MT possesses defense properties against biotic stresses, enhancing the plant ability to respond to these challenges. Similarly, several studies have highlighted the vital role of MT in mediating plant responses to pathogens, revealing its potential as a protective agent in plant defense mechanisms (**Figure 3**).

### 5.1 Antifungal properties of MT

Exogenous MT enhances plant resistance to biotic stress by improving plant tolerance to infections caused by fungal pathogens. For example, MT attenuates fungal infections in cotton (Li et al., 2019a) and enhances resistance to yellow wilt by influencing lignin and cotton phenol synthesis genes in the phenylpropane, MVA, and cotton phenol pathways. MT promotes resistance to gray mold in tomato fruits by regulating H_2_O_2_ production and the jasmonic acid signaling pathway (Liu et al., 2019a). Spraying exogenous MT on potato leaves and tuber slices significantly reduces late blight by suppressing mycelial growth, modifying cellular ultrastructure, and lowering stress tolerance of pathogenic blight mold (Zhang et al., 2017b). MT treatment resulted in a decrease in both the rate of contamination and the number of pathogenic fungal sporocarps in *Arabidopsis thaliana*. This reduction was attributed to the increased expression of PR3 and PR4 genes, which are responsive to JA (Moustafa-Farag et al., 2020). Several theories explain how the MT prevents fungal infections in plants. For example, some scientists suggest that MT defense mechanism involves its ability to maintain cellular H_2_O_2_ concentrations and regulate antioxidant enzyme activities (Aghdam and Fard, 2017a).

### 5.2 Antiviral properties of MT

Exogenous MT has been investigated to reduce viral infections in post-harvest fruits. For instance, exogenous MT caused post-harvest grapevine resistance to ashwagandha by activating the defense response (Li et al., 2022c). In blueberries, MT increases postharvest disease resistance by mediating the phenylpropane and jasmonic acid signaling pathways, thereby effectively controlling decay during storage (Qu et al., 2022). Furthermore, exogenous MT treatment has been discovered to increase postharvest disease resistance in tomatoes (Jiping et al., 2020), strawberries (Promyou et al., 2023), and papayas (Fan et al., 2022). This increased resistance is primarily attributed to the MT role in scavenging ROS, boosting antioxidant capacity, preserving metabolic energy balance, and enhancing the activity of defense-related enzymes. Another study revealed that MT significantly decreased DNA methylation and changed gene expression in grape berries, improving flavonoid biosynthesis and disease resistance by lowering the methylation level of related gene promoters (Gao et al., 2020).

MeRAV1 and MeRAV2 were found to positively regulate seven MT biosynthesis genes and endogenous MT levels in cassava experiments. In addition, MeRAV1 and MeRAV2 are direct transcriptional activators of three cassava MT biosynthesis genomes (MeTDC2, MeT5H, and MeASMT1), and they both positively regulate plant resistance to cassava leaf blight (Wei et al., 2018). Furthermore, the action of MT and selenium together activated antioxidant enzymes and upregulated PR protein gene expression, potentially strengthening tomato fruit defenses against the post-harvest pathogenic fungus gray mold (Zang et al., 2022). Overall, MT has a critical role as a signaling molecule in plant stress response. It acts as an effective antioxidant, scavenging the large accumulation of ROS/RNS caused by a range of environmental stresses, thereby enhancing the activity of the antioxidant defense system and improving plant resistance.

This chapter summarizes the functions of MT under different biotic stresses. Plants will activate their immune system when subjected to biotic stresses to recognize pathogens and protect themselves through different pathways. MT plays an important role in the plant response to these pathogens, enhancing tolerance and preventing damage caused by infections. In addition, MT has demonstrated resistance to postharvest fruit viruses, improving fruit resilience by activating the defense response system, which helps prolong the storage period of fruits. Furthermore, MT acts as a signaling molecule that regulates plant resistance to viruses, thereby enhancing their overall defense capabilities.

## 6 Melatonin crosstalk with other plant hormones

### 6.1 Melatonin and Abscisic Acid

Abscisic acid (ABA) is a key phytohormone that controls numerous physiological activities, such as stomatal opening and seed germination. Due to its dual nature, ABA acts both as a promoter and an inhibitor of plant growth (Parwez et al., 2022). MT and ABA are crucial plant hormones that play critical roles in plant growth and development. Under unfavorable conditions, the accumulation of ABA usually inhibits plant growth and development, whereas MT promotes these processes. The interaction between these two factors controls plant growth throughout its developmental stages (**Table 2**). Mansouri et al. (2023a) revealed that the exogenous application of MT (10 µM) reduced endogenous ABA levels, influencing the expression of several genes involved in fruit ripening (CHS, GAMYB, SnRK2.6 and PAL) and inhibiting endogenous ABA signaling to delay strawberry fruit ripening. Additionally, exogenous MT treatment inhibits the expression of genes involved in the ABA pathway and accumulates ABA, which delays the leaf senescence and enhances drought resistance (Li et al., 2015).

**Table 2:** Studies on the interaction of different concentrations of MT with plant hormones.

Crop Plant	Plant hormone	Application method	Dose of MT for treatment	Effect	References
Apricot	GA	spray	MT/GA(25mg/L)	Promotes nutrient growth and improves *growt*h, fruit set, yield and fruit quality	(Saied Kamel Mohamed Abd et al., 2023)
Rapeseed	GA	soak seeds	GA(500 mg/L) 、MT(500µM)	Scavenging ROS and more proline accumulation	(Khan et al., 2020b)
Cucumber seedlings	GA	soak seeds	MT(1µM)	Up-regulation of GA biosynthesis genes and increased GA content improve germination rate	(Zhang et al., 2014a)
Cotton	ABA	spray	MT(200µM) 、ABA(0.5µM)	Regulates antioxidant enzyme activity, scavenges ROS, increases lint yield, and improves ascorbate peroxidase activity	(Hu et al., 2021b)
Apple	ET	spray	MT(1mM)	Reduced ethylene content and modulation of antioxidant enzyme activity	(Onik et al., 2021)
Tomato fruit	ET	immerse	MT(50µM)	Promoting lycopene accumulation and tomato fruit flavor	(Sun et al., 2020)
Strawberry fruits	ET	immerse	MT(100µM)	Slows post-harvest aging and increases antioxidant to extend shelf life	(Aghdam and Fard, 2017a)
Wheat	SA	foliar spray	MT(70µM)/SA(75mg/L)	Increased photosynthetic pigment, photosynthetic rate and stomatal conductance	(Talaat and Nutrition, 2021)
Tomato (in Chromium stress)	JA	foliar spray	MT(100μM) 、 JA(5μM)	Reduced nitrogen metabolizing enzyme activity induced by chromium toxicity, up-regulated antioxidant system functions	(Qin et al., 2024)
Brassica juncea roots	IAA	rooting	MT(0.01-0.5µM)	Increase IAA content and promote root growth	(Chen et al., 2009)
Creeping bentgrass	CK	foliar spray	MT(20µM)	Reduction of enzyme activity and genes that break down chlorophyll to prevent leaf senescence	(Ma et al., 2018)

GA, gibberellic acid; ABA, abscisic acid; ET, ethylene; SA, salicylic acid; JA, jasmonic acid; IAA, auxin; CK, cytokinin.

There are synergistic effects between MT and ABA, for example, exogenous MT and ABA can both reduce the oxidative damage caused by cold stress. Pretreatment with exogenous MT improves cold tolerance by inducing the production of endogenous MT, which may act as a second messenger to activate the downstream cold-responsive genes (EnCBF9, EnCBF14, and EnCOR14a). This attenuates the accumulation of ROS and strengthens the antioxidant defense system (Fu et al., 2017). Under low-temperature stress, irrigating cucumber seedlings with 200 µM MT solution significantly attenuated low-temperature induced injury, which was attributed to the up-regulation of the stress-related gene CsZat12 by MT, the increase of putrescine, spermidine and stabilized spermine by altering polyamine metabolizing enzyme activities, and finally, the MT modulation of ABA biosynthesis genes (CsNCED1 and CsNCED2) alongside the expression of ABA catabolic genes (CsCYP707A1 and CsCYP707A2) to alleviate cold stress in cucumber (Zhao et al., 2017). Li et al. (2016) concluded that the application of spraying exogenous MT at the four-leaf stage of wheat enhanced drought priming induced cold tolerance (DPICT) by modulating the subcellular antioxidant system and ABA levels in barley, as presented in their study of cold hardiness in two barley species.

Under drought stress, exogenous MT and ABA usually exhibit negative crosstalk mechanisms. MT protects apple plants from drought and ABA damage by selectively upregulating catabolic genes (MdCYP707A1 and MdCYP707A2) and downregulating ABA synthesis (MdNCED3) (Li et al., 2015). Similarly, foliar spraying of MT (100 µM) promoted stomatal behavior and gas exchange in drought-stressed maize seedlings by blocking the ABA synthesis gene NCED1 and inducing ABA catabolic genes (ABA8ox1 and ABA8ox3) (Li et al., 2021). Exogenous MT also increases cucumber seed germination in salt stress situations by reducing ABA accumulation (Zhang et al., 2014a). The application of MT, ABA and MT+ABA under salt stress revealed a significant up-regulation of MT biosynthesis gene expression, an increase in ABA catabolism and a down-regulation of anabolic metabolism, leading to a decrease in the endogenous ABA content in tomato seedlings. Despite the different key pathways that each treatment employs to cope with salt stress, the MT+ABA treatment was particularly effective in promoting the growth of tomato seedlings under salt stress (Hu et al., 2021a). When the first node of the fourth fruit branch blossomed simultaneous spraying of MT and ABA on cotton can promote the enzymatic antioxidant system (up-regulation of Cu/ZnSOD, MnSOD) and the ASC-GSH cycle. This enhancement facilitates the scavenging of ROS, enhance the hydration status and antioxidant capability of drought-stressed leaves, which will ultimately lead to a rise in lint cotton output (Hu et al., 2021b).

Leaf senescence may be a natural process of plant aging that is accelerated by abiotic stresses. However, ABA and MT play opposite roles in regulating this process. The negative crosstalk mechanism of MT inhibition of ABA biosynthesis and signaling is particularly important in the regulation of plant senescence as well as fruit ripening. For example, exogenous MT application reduced the activity of genes responsible for ABA production and slowed leaf aging in cabbage (Tan et al., 2019b). Mansouri et al. (2021) found that injection of 1000 µM MT into strawberries delayed fruit ripening by boosting the genes involved in MT biosynthesis and inhibiting endogenous ABA signaling.

### 6.2 Melatonin and Ethylene

The gaseous plant hormone ethylene (ET) was the first hormone to be identified. It is the most basic olefinic gas biosynthesized by plants, it regulates plant growth, development, and stress responses through well-established signaling pathways (Binder, 2020). MT controls several ripening factors (RIN, CNR, NOR and AP2a) by triggering ethylene production. During tomato ripening, soaking the fruits in 50 µM MT enhanced the expression level of genes associated with ET, resulting in increased fruit softness, and water-soluble pectin. As a result, plants accumulated 22.5% higher water-soluble pectin and 19.5% lower protopectin compared to controls. MT also upregulated the level of 1-aminocyclopropanecarboxylic acid synthase 4 to increase ET production, which contributes to tomato fruit ripening and quality improvement (Sun et al., 2015). This experiment demonstrated that there is some synergy between MT and ET in regulating tomato fruit ripening.

Other experiments involving exogenous MT applied prior to fruit ripening also showed a synergistic interaction between MT and ET in regulating fruit ripening. For example, 10-100 µM MT significantly increased ABA and ET content and encouraged fruit ripening in grapes (Xu et al., 2018). Verde et al. (2022) found that MT treatment significantly promoted ET production, stimulated fruit ripening, improved the quality of ripening apple fruits.

Postharvest MT application can improve postharvest fruit quality. For example, exogenous MT stimulates ET biosynthesis and increases ethylene availability through increasing the MdACS1 and MdACO1 genes transcriptional levels, contributing to improved fruit quality during postharvest ripening (Verde et al., 2023). At this stage a cooperative crosstalk mechanism between MT and ET is also observed. However, two other experimental studies have found a negative feedback regulation between the two hormones. MT attenuated the yellowing of postharvest kale-type oilseed rape leaves and prolonged its shelf-life by inhibiting the production and action of ET. MT-treated plants significantly blocked the ET biosynthesis pathway by decreasing the expression level and activity of 1-aminocyclopropane-1-carboxylate synthase and ACC oxidase (Wang et al., 2022c). MT and nitric oxide (NO) play important roles in reducing the up-regulation rate of flesh softening-related genes (PcCel and PcPG), inhibiting the level of ET synthase genes (PcACS and PcACO), and decreasing the respiration and ET production rates, thereby delaying pear fruit senescence (Liu et al., 2019b).

Recent studies have found that a negative crosstalk mechanism between MT and ET can increase plant resistance to environmental stress. For example, applying MT and aminoethoxyvinylglycine (AVG, an ethylene biosynthesis inhibitor) enhanced photosynthesis and antioxidant capacity of grape leaves, suggesting that MT may reduce O_3_ stress by suppressing ET biosynthesis (Liu et al., 2021). In another study, MT-mediated salt tolerance was associated with the inhibition of ET synthesis. Wheat plants treated with AVG (50 µM) and MT (100 µM) could maintain photosynthesis and plant dry weight by regulating GR activity, GSH and cellular ET levels under salt stress conditions (Khan et al., 2022).

### 6.3 Melatonin and Auxin

**Figure 4:**
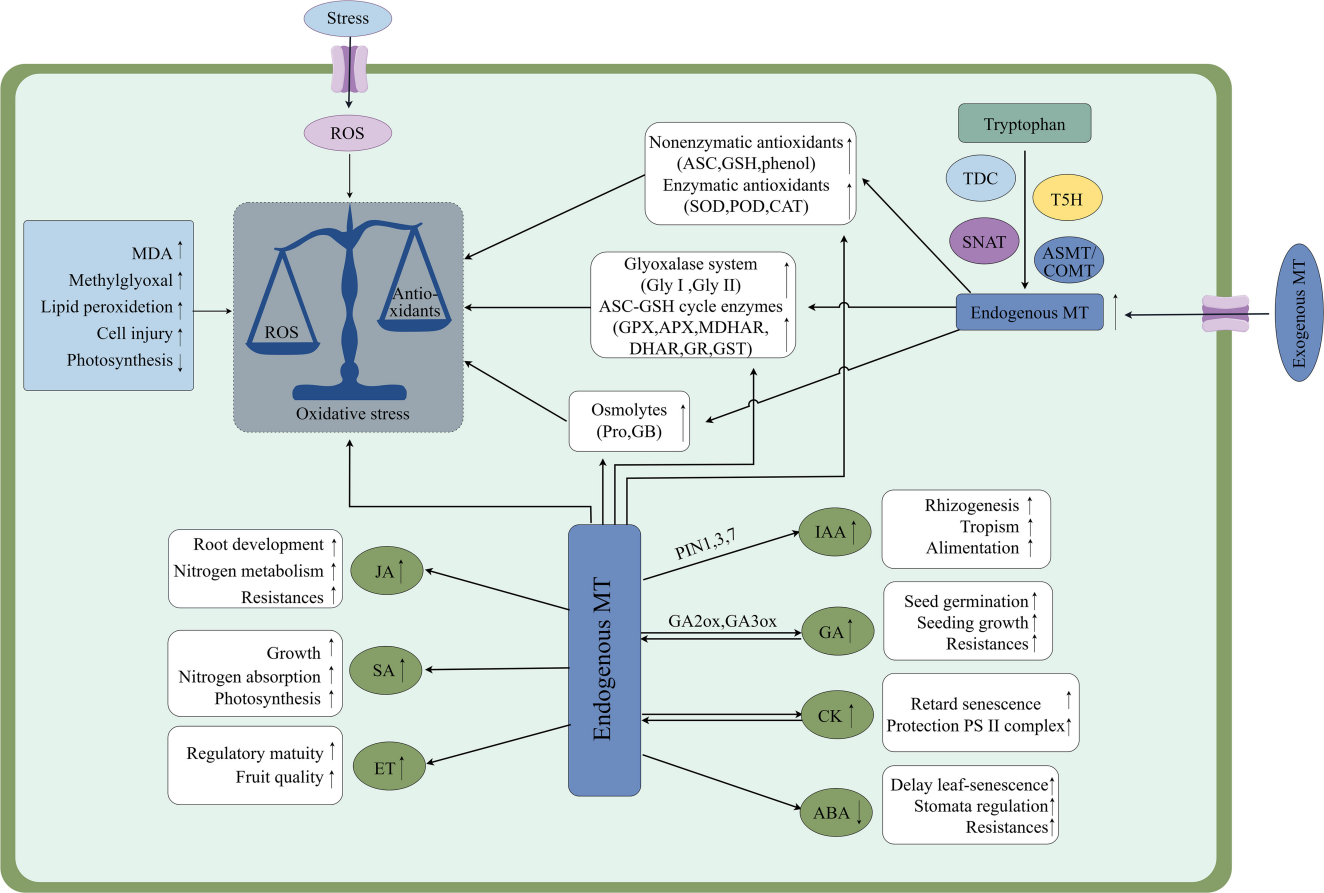
Effects of melatonin-mediated signaling on abiotic stress in plants and exogenous MT promotes endogenous MT production through up-regulation of its biosynthesis-critical enzyme activities, and interactions with other phytohormones to counteract damage caused by abiotic stress. MT, Melatonin; MDA, Malondialdehyde; ASC, Ascorbic acid; GSH, Glutathione; Pro, Proline; GB, Glycine betaine; SOD, Superoxide dismutase; POD, Peroxidase; CAT, Catalase; Gly-I, Glyoxalase I; Gly-II, Glyoxalase II; ASC-GSH, Ascorbate-glutathione; GPX, Glutathione peroxidase; MDHAR, Monodehydroascorbate lyase reductase; APX, Ascorbate peroxidase; DHAR, Dehydroascorbate reductase; GR, Glutathione reductase; GST, Glutathione S-transferase. Symbol ↑ indicates an upward adjustment.

Auxin (IAA) is a large class of phytohormones that serves as major regulators of plant growth, mainly through the control of cell division, elongation and differentiation. Studies have demonstrated that both MT and growth hormone 3-indoleacetic acid (IAA) are indole compounds, that share the same biosynthetic precursor, tryptophan (Trp), and have similar conformations (Wei et al., 2022). There is growing evidence that MT is a growth hormone-like molecule, promoting nutrient uptake and stimulating growth and root development, especially in adventitious and lateral roots. For instance, MT synergizes with IAA to enhance lateral root development in wild-type Arabidopsis by regulating growth hormone distribution through modulation of IAA transport (Ren et al., 2019). The literature suggests that there is an interaction between MT and IAA signaling, with MT promoting IAA accumulation by upregulating genes involved in IAA synthesis in tomatoes (IAA19 and IAA24) (Wen et al., 2016). For example, the application of exogenous MT to soybean plants not only up-regulated several YUCCA genes involved in IAA biosynthesis but also improved the expression of genes encoding IAA receptors (TIR1, AFB3 and AFB5) (Wei et al., 2022). MT also regulates IAA transport in plants. It was shown that MT significantly down-regulated five genes (LAX2, PIN5, TT4, TT5, and WAG1) encoding IAA transporter proteins or proteases involved in the IAA regulation in Arabidopsis. significant downregulation suggests that MT play a role in fine-tuning intracellular distribution of IAA, thereby affecting lateral root development (Binder, 2020). Under drought conditions, IAA levels in maize leaves showed a transient decreasing trend between the V-9 and R-3 growth stages, and exogenous MT significantly increased indole acetic acid levels in stressed leaves and restored plant growth. For example, 100 µM MT applied as a soil infiltrate to roots enhanced IAA levels by 31.71%, 38.40%, and 34.90% in maize at the V-9, R-1 and R-3 growth stage compared to the drought-stress controls (Ahmad et al., 2022). In addition, there is an important relationship between MT and IAA in regulating plant salt stress. Miao et al. (2024) found that double mutants with attenuated MT synthesis exhibited salt sensitivity, and the transcriptome analysis showed that the expression of a large number of salt-responsive genes was affected by SNAT defects, which were associated with IAA biosynthesis and various signaling pathways. This suggests that endogenous MT participates in plant salt response through affecting IAA signaling. As essential regulators of plant growth, MT and IAA interact with other hormonal systems to impact IAA production, transport, and signaling, which in turn impacts every facet of plant physiology (**Figure 4**) (Sun et al., 2021).

### 6.4 Melatonin and Cytokinin

Cytokinin (CK) are a group of plant hormones that play an important role in regulating plant growth and stress responses. They are involved in different developmental and physiological functions of plants, including cell division, shoot formation, seed germination, leaf senescence, and promotion of plant growth.

Under unfavorable environmental conditions, there are synergistic crosstalk mechanisms between MT and CK. For instance, MT upregulates the CK biosynthesis genes (LpIPT2 and LpOG1) in response to heat stress. In addition, the response transcription factors B-ARR and A-ARR in the CK signaling pathway are up- and down-regulated. Although MT did not alter CK levels compared to non-stress conditions, this suggests that exogenous MT increased endogenous MT and CK levels in plant tissues (Zhang et al., 2017a). Yu et al. (2022) found that MT scavenges ROS by repressing the expression of the senescence-related gene SlCV. As a result, MT stabilizes the chloroplast architecture and delays leaf withering, while attenuating the interactions among SlCV and SlPsbO/SlCAT3, which decreases ROS generation and enhances ROS removal in photosystem II. Similar to MT, CK also slows down the senescence process. Transcriptome analysis and endogenous hormone assays showed that CiS40-11 inhibits leaf senescence by advancing cytokinin peptide biosynthesis through blocking the expression of AtMYB2 in CiS40-11 overexpression lines (Yang et al., 2022). Exogenous MT and CK together inhibit drought-induced leaf senescence by maintaining chlorophyll content (through down-regulation of chlorophyll catabolic metabolism genes), enhancing photochemical efficiency, preserving relative water content, and improving physiological traits in terms of reduced H_2_O_2_ content, lipid peroxidation and electrolyte leakage.

Under drought conditions, CK and MT acted synergistically in transgenic creeping bentgrass overexpressing isopropenyltransferase (ipt). Exogenous MT also significantly up-regulated the expression of ipt and CK signaling genes in ipt transgenic plants, which synergistically regulated the plant response to drought stress (Ma et al., 2018). In addition, MT synergizes with CK under high light (HL) conditions in Arabidopsis. MT reduces photodamage and aids in the recovery of CK synthesis genes (LOG7, IPT3 and IPT5), and signaling genes (AHK2,3 、ARR 1,4,5) (Bychkov et al., 2023). Recent studies indicate that MT regulates primary root growth in Arabidopsis partly through a growth hormone-dependent pathway that synergizes with CK (Yanping et al., 2022). The mediator proteins involved in the molecular crosstalk between MT and CK have been unraveled by Sliwiak et al. (2018). Researchers have identified the pathogenesis-related class 10 (PR-10) protein LIPR-10.2B isolated from yellow lupin (*Lupinus luteus*), which has a binding affinity for Mel and trans-zeatin. This unique complex provides insights into the role of PR-10 proteins in phytohormone regulation, suggesting that PR-10 proteins are low-affinity melatonin binders in the presence of elevated melatonin concentrations (Sliwiak et al., 2018). Thus, MT and CK participate in a synergistic crosstalk mechanism under unfavorable environmental conditions.

### 6.5 Melatonin and Gibberellic Acid

Gibberellic Acid (GA) is a key regulator of plant developmental and growth processes. Its primary roles are to regulate phytohormone signaling, accelerate internode elongation and leaf expansion, and control seed germination, stem elongation, and floral development. MT affects and the activity of GA-responsive genes to regulate the complex processes of GA biosynthesis and metabolism in plants. For example, exogenous MT can up-regulate GA biosynthesis genes (GA20ox and GA3ox) in plants to improve GA accumulation and encourage the establishment of cucumber seedlings under salt stress (Zhang et al., 2014b). Meanwhile, exogenously applied GA can also stimulate endogenous MT in plants. For example, exogenous gibberellin 3 (GA3) treatment significantly induced MT synthesis in rice seedlings, while the GA synthesis inhibitor dobutazole severely reduced MT synthesis. This suggests that GA treatments can be used for the production of melatonin-rich seeds, vegetables, and fruits (Hwang and Back, 2022).

Under salt stress, MT has been shown to promote the development of oilseed rape seedlings by raising the transcript count of GA biosynthesis genes (LOC106442380, LOC106398539, LOC106357323) and repressing the inhibitory activity of DELLA on GA signaling (Tan et al., 2019a). The combined application of GA (1.4 µM) and MT (100 µM) reduces MG levels by activating the activity of the glyoxalase machinery (Gly-I and Gly-II), effectively inhibiting excess salt-induced ROS development. The synergetic benefits of GA and MT have been shown in tomato plants, where they enhanced the amounts of antioxidant enzymes (CAT and GR) and osmoregulatory compounds (Pro and GB) to preserve redox homeostasis and promote tomato seedling growth in salt-stressed environments (Manzer et al., 2020). Seed induction by exogenous MT and GA increases proline accumulation for osmoregulation and activates several non-enzymatic antioxidants to scavenge drought-induced excess ROS production, thereby increasing the redox potential, which is essential for improving yield and yield-related parameters in rapeseed under drought stress (Khan et al., 2020a).

MT stimulation of seed germination was also linked to increased GA expression. Specifically, exogenous MT positively upregulates GA biosynthetic genes (GA2ox and GA3ox), significantly increasing seed GA (especially GA4) content, and promoting cucumber germination under high salinity (Zhang et al., 2014a). Likewise, rapeseed pretreated with GA3 and MT maintained seed yield and quality under drought by increasing antioxidant enzymes (SOD, POD, and CAT), along with GSH and ASC contents (Khan et al., 2020b). There is a direct association and a positive crosstalk relationship between MT and GA that plays a significant role in tomato seedlings to promote heat tolerance (Jahan et al., 2021b).

Overall, the common effects of MT and GA on plants are primarily showed within the advancement of plant growth and enhancement of stress tolerance. They act synergistically through different mechanisms to promote plant growth.

### 6.6 Melatonin and Salicylic Acid

The phytohormone salicylic acid (SA) is a b-hydroxyphenolic acid that aids plant defense. SA and MT have some synergistic effects in resisting environmental stresses, particularly in mitigating salt stress. For instance, the combined application of SA and MT promotes wheat growth and productivity by attenuating salinity-induced oxidation through reduced photosynthetic inhibition and improved carbon assimilation (Talaat and Nutrition, 2021). Similarly, SA and exogenous MT treatments could mitigate the negative effects of salt stress on wheat growth and yield by increasing the metabolic flux of polyamines, thereby promoting polyamine biosynthesis. Improved nitrogen uptake may be responsible for the higher plant performance following MT and SA applications, as these treatments up-regulate the activities of nitrogen metabolism-related enzymes and increase nitrogen uptake, thereby improving the photosynthetic capacity of the plant (Neveen, 2021). Talaat and Shawky, 2022 discovered that wheat plants treated with SA+MT had considerably higher nutrient contents (N, P, K^+^, Fe, Zn and Cu) compared to untreated plants under salt stress conditions. In addition, SA+MT treatment significantly reduced cellular Na^+^ accumulation and H^+^ pumping activity in roots, thus minimizing the negative effects of salt on wheat (Talaat and Shawky, 2022).

Yan et al. (2023) showed that spraying *Hibiscus sabdariffa* plants with MT and SA promoted the recovery of chlorophyll content under drought stress. This growth recovery was achieved by MT+SA induced up-regulation of drought-related genes in leaf tissues (Yan et al., 2023). In drought-stressed tomato plants, MT+SA up-regulated the glyoxalase system to limit methylglyoxal production, which significantly improved leaf properties such as chlorophyll content, Fv/Fm ratio, relative water content and biomass assimilation (Kaya et al., 2023).

Studies suggests that using MT and SA can protect plants from heavy metal toxicity. There is a synergistic effect between the two in mitigating heavy metal stress. For example, MT+SA protected sword lily plants from arsenic toxicity by boosting the activity of antioxidants enzymes, proteins, and proline levels (Zulfiqar et al., 2023). Similarly, MT and SA co-treatment increased tolerance to arsenic toxicity by limiting arsenic ions in pepper tissues (Kaya et al., 2022). In addition to arsenic, cadmium contamination is now a major research topic and both exogenous MT and SA at 100 µM reduced cadmium uptake, improved chlorophyll biosynthesis, and modulated the ascorbate-glutathione and glyoxalase systems to protect safflower seedlings from cadmium toxicity (Amjadi et al., 2021). Furthermore, MT has been reported to increase the accumulation of SA and increase the expression of defense genes (SlWRKY70, SlNPR1, SlTGA5, SlPR1 and SlPR2) in tomato, providing protection against gray mold damage (Liu et al., 2019a).

### 6.7 Melatonin and Jasmonic Acid

Jasmonic Acid (JA) is a class of oxygenated lipid derivatives that are significant for plant growth and environmental adaptation due to their specific regulatory functions. JA plays various regulatory roles in plant growth, such as affecting seed germination, axial elongation, root organ formation, flower growth, stomatal regulation and leaf senescence (Huang et al., 2017). In addition, it plays a crucial role in mediating plant responses and defenses against environmental stressors, thereby increasing plant resistance to various stresses. For instance, rice plants sprayed with 400 µM JA showed up-regulation the expression of defense-related genes (OsPR10a, OsAOS2), while down-regulating the OsEDS1 gene, thereby effectively controlling rice blast disease (Wang et al., 2021b).

MT and JA show synergistic effects in certain plant regulatory processes, particularly in enhancing cold resistance. Under cold stress, higher accumulation of JA strongly induces the expression of two MT biosynthesis genes, SlSNAT and SlAMST, leading to higher MT biosynthesis. The positive feedback loop between MT and JA biosynthesis enhances the plant ability to withstand cold conditions (Ding et al., 2021). Transcriptomic analysis revealed 957 differentially expressed genes in drought-exposed maize seedlings with and without MT, indicating that the mutual crosstalk between MT and JA is necessary for enhancing drought tolerance in maize seedlings (Zhao et al., 2021). Similarly, exogenous application of MT (100 µM) increased JA content in wheat seedlings under drought stress, enhancing drought tolerance by upregulating the expression of JA biosynthesis genes (LOX1.5 and LOX2.1) and transcription factors (HY5 and MYB86) (Luo et al., 2023).

Under biotic stress conditions, such as gray mold infection, applying MT (50 µM) increased the activities of antioxidant- and defense-related enzymes to trap excess H_2_O_2_ and induce the JA signaling pathway. Meanwhile, MeJA application up-regulated the expression of SlLoxD, SlAOC and SlPI II while decreased the expression of SlMYC2 and SlJAZ1 (Liu et al., 2019a). MT also induced genes related to JA (VaLOX, VaAOS, and VaAOC), pathogenesis-related proteins (VaGLU and VaCHT), as well as phenylpropane metabolism (VaPAL, VaC4H, Va4CL, VaCAD, VaPPO, and VaPOD) to promote postharvest resistance in blueberry fruits (Fan et al., 2018a). The simultaneous application of MT and JA can protect plants from heavy metal damage. Qin et al. (2024) found that foliar sprays of JA and MT promoted the activities of nitrogen metabolizing enzymes in Cr-stressed tomato plants. The antioxidant system was upregulated to reduce oxidative damage caused by metals, increasing secondary metabolites and phenylalanine deaminase activity. However, negative feedback regulation has also been found. In rapeseed seedlings, the application of MT under saline conditions negatively affected JA biosynthesis and concentration. This inhibition was mainly mediated by up-regulation of hydroperoxide lyase 1 (HPL1) and down-regulation of alkenyl oxide cyclase (AOC), as well as in increase in the production of JAZ proteins (which acted as negative regulators of JA signaling), thereby, inhibiting the transcriptional activity of JA (Tan et al., 2019a).

## 7 Conclusion and future prospects

MT, as a multi-effector molecule, has received extensive attention in recent years for its role in plant growth and development. When plants face adversity MT alleviates the stress caused by abiotic and biotic factors by specifically scavenging the ROS produced during stress. This process enhances the antioxidant capacity of the crop, reducing the cellular damage caused by free radicals and improving photosynthesis. Endogenous MT can be synthesized at different sites within the plant and distributed throughout different parts of its body. However, endogenous MT alone may not sufficiently protect plants from severe stress, therefore, exogenous MT has shown significant coping effects in unfavorable environments by promoting growth regulation, slowing leaf senescence, enhancing photosynthesis, and improving the antioxidant system that scavenges ROS. In addition to MT, other plant hormones such as IAA, CK, and ABA also improve environmental adaptability by improving the physiological and metabolic processes under stressful conditions. Under stress conditions, the combined application of melatonin with hormones such as gibberellin, cytokinin, abscisic acid and salicylic acid has been proven to be more effective that using melatonin alone. For instance, during drought stress, the synergistic effect of combining melatonin and gibberellin helps maintain redox homeostasis and promotes osmotic pressure regulation, thereby reducing salinity-induced oxidative stress and protecting seedlings from ROS damage. Melatonin and cytokinin can reduce enzyme activity and gene expression that decompose chlorophyll under drought stress, preventing leaf senescence. Additionally, the combined application of melatonin and abscisic acid can increase the relative water content and total antioxidant capacity of plant tissues in leaves. This synergistic approach improves the ability of reduced ascorbic acid to eliminate ROS and boosts the expression of salt-regulating genes. Furthermore, the combined treatment of melatonin and salicylic acid significantly increases the acquisition of nitrogen, phosphorus, potassium and trace elements under drought stress.

This article reviews the interactions between MT and various hormones such as IAA, CK, GA, ABA, ET, SA, and JA based on existing research. It analyzes the roles of synergistic and antagonistic hormone interactions in controlling the growth, development, and stress response of plants, among other physiological processes. At present, some progress has been made in understanding the synthesis pathway of MT, the regulatory mechanism underlying this complex pathway is still unclear. The appropriate concentration and duration of MT application are significant factors in regulating plant growth. Further research is required to determine the optimal concentration and timing of MT for specific plants in different adverse environments. The outstanding performance of MT in abiotic and biotic stress requires further exploration of its mechanism in larger scale field experiments in complex environments, as well as the promotion of its application in future research. In the fight against biological stress, it is urgent to explore the synergistic effects of multiple pest and disease treatment methods, alongside the application of insecticides and fungicides, to achieve sustainable environmental development. The interactions of MT with other hormones, as well as the mediating mechanism that enhance plant tolerance, are still unclear. One of the meaningful future studies is the physiological mechanism and core position of MT in directing hormone homeostasis in plants, particularly regarding the potential synergistic or antagonistic effects of MT and other hormone-mediated responses.
